# When Mindfulness Interacts With Neuroticism to Enhance Transformational Leadership: The Role of Psychological Need Satisfaction

**DOI:** 10.3389/fpsyg.2018.02588

**Published:** 2018-12-18

**Authors:** Anouk Decuypere, Mieke Audenaert, Adelien Decramer

**Affiliations:** Department of Human Resource Management and Organizational Behavior, Faculty of Economics and Business Administration, Ghent University, Ghent, Belgium

**Keywords:** transformational leadership, leader mindfulness, psychological need satisfaction, neuroticism, self-regulation, emotion-regulation

## Abstract

Transformational leadership is a popular and well-researched leadership style. Although much is understood about its positive consequences, less research has focused on antecedents of transformational leadership. In this research we draw upon self-determination theory and incorporate a self-regulatory approach to investigate if and how leader mindfulness influences transformational leadership. The analyses show that autonomy, competence and relatedness need satisfaction mediate between mindfulness and transformational leadership, indicating that mindfulness is associated with psychological need satisfaction. Furthermore, the data show that neuroticism moderates the relationship between mindfulness and relatedness need satisfaction. Generally speaking, the association between mindfulness and relatedness need satisfaction is positive. When neuroticism is also high, mindfulness has the largest impact. Or conversely, when emotional stability is high, mindfulness has the smallest association with relatedness need satisfaction. This is in line with evidence suggesting that mindfulness may primarily exert its influence through emotional self-regulation. Furthermore, the moderated mediation model for relatedness need satisfaction is significant, indicating that neuroticism is a boundary condition for the indirect effect of mindfulness on transformational leadership through relatedness need satisfaction.

## Introduction

Transformational leadership is a well-known and well-researched leadership style (Bass and Avolio, 1990). It has been related to a number of outcomes including innovation (Kraft and Bausch, 2015), organizational commitment and citizenship behavior, job performance (Zhu et al., 2013), job satisfaction, team performance (Braun et al., 2012), and trust (Judge and Piccolo, 2004). In addition, since today's world is characterized by volatility, uncertainty, complexity, and ambiguity (Rodriguez and Rodriguez, 2015), the role of leaders to provide guidance in these turbulent times is more important than ever. Transformational leaders fulfill this role since they envision a future, act as a role model, set performance standards, show determination and confidence, and are described as being able to transform interactions from “pure self-interest to having interest for others” (Kopperud et al., 2014, p. 3). In sum, transformational leaders aim at transforming employees' mindsets toward achieving organizational goals (Bass and Avolio, 1990). Transformational leaders are characterized by (1) idealized influence, i.e., leader charisma and making employees feel good, (2) intellectual stimulation, i.e., stimulating creativity and innovation, (3) inspirational motivation, i.e., providing a vision, and (4) individualized consideration, i.e., considering each employee individually and taking into account individual differences.

Although there has been much research investigating the consequences of transformational leadership for both employees and organizations, “little is known about the social and motivational factors that influence transformational leadership behavior” (Trépanier et al., 2012, p. 272). This is not to say that scholars on transformational leadership have neglected research on antecedents completely: research has investigated e.g., the role of cynicism about organizational change (Bommer et al., 2004), peer leader behavior relationships (Bommer et al., 2004) and leaders' workplace relationships (Trépanier et al., 2012) or leader's emotion recognition and personality (Rubin et al., 2005). Nevertheless, the actual mindset of leaders, and the way in which they pay attention, has been less scrutinized (Sauer and Kohls, 2011). There is a need for research to investigate a possible pathway through which transformational leaders may perform better in our ever-changing, “VUCA,” world (Rodriguez and Rodriguez, 2015).

In order to address this need, we study mindfulness, which can be defined as a way to pay attention in a particular way: intentional, in the present moment and non-judgmental (Kabat-Zinn, 1994). Research has shown that mindfulness in an organizational context is related to reduced emotional exhaustion (i.e., the core component of burn-out; Reb et al., 2016), more job satisfaction (Hülsheger et al., 2013), more authentic functioning and work engagement (Leroy et al., 2013), better decision making through reducing bias (Karelaia and Reb, 2015) and better performance (Reb et al., 2016). A recent meta-analysis on mindfulness interventions on the work floor has indicated that short interventions may be a valuable tool for managing psychological distress (Virgili, 2015). In sum, mindfulness has been shown to reduce stress and enhance well-being. Since previous research on transformational leadership has shown that a leader's happiness and well-being contribute to transformational leadership (Jin et al., 2016), this study will examine whether mindfulness may help increase a leaders' psychological need satisfaction and consequently enhance transformational leadership.

To date, only one research study we know of linked mindfulness to transformational leadership (Pinck and Sonnentag, 2017). In this research, it was shown that transformational leadership mediates between leader mindfulness and employee well-being. We add to this work by investigating exactly how mindfulness is related to transformational leadership. Mindfulness may be beneficial for transformational leaders in several ways. In general, since mindfulness enhances general functioning through emotion regulation, enhancing focus and work engagement(Brown and Ryan, 2003), it should help leaders to perform acts in accordance with positive leadership styles. In addition, mindfulness should help facilitate “attentive, stimulating, and inspiring behavior that characterizes transformational leadership” (Pinck and Sonnentag, 2017; p. 2).

We draw mainly on self-determination theory and a self-regulatory approach to further theorize how and under which circumstances mindfulness leads to transformational leadership. Based on self-determination theory (SDT; Deci and Ryan, 2008; Van Den Broeck et al., 2008a), we argue that psychological need satisfaction is one of the possible underlying processes that explains how leader mindfulness impacts transformational leadership. SDT states the need for autonomy, competence and relatedness represent the fundamental nutrients that are necessary for growth, integrity, and well-being (Deci and Ryan, 2000). These basic needs are proposed to drive an autonomous, even intrinsic, motivation at work (Deci and Ryan, 2008; Van Den Broeck et al., 2008a). Psychological need satisfaction has been related to well-being, health, and performance (Deci and Ryan, 2000). It is plausible to assume that leaders whose psychological needs are fulfilled, will be more energized to perform transformational behaviors that go above and beyond their managerial job description. Furthermore, since the influence a leader has on his/her own psychological need satisfaction depends on their mindset, decision making and self-regulation, mindfulness may be one of the key drivers of this process (Glomb et al., 2011; Hülsheger et al., 2013). More specifically, mindfulness may impact the three psychological needs in different ways (see “The mediating role of psychological need satisfaction” below). When leaders feel well-through mindfulness and psychological need satisfaction, they have more resources to perform exceptional transformational leader behaviors. This assumption is also in line with predictions for the conservation of resources theory (Hobfoll, 2001), which proposes that the loss of (personal) resources leads to stress. Since mindfulness helps to manage stress, and therefore conserve cognitive resources, it might ensure that energy levels remain sufficiently high to perform transformational leader behaviors. We propose that this may be especially true for transformational leadership, based on the reasons outlined below that specify exactly how mindfulness can lead to psychological need satisfaction and benefit transformational leadership.

**Figure 1:**
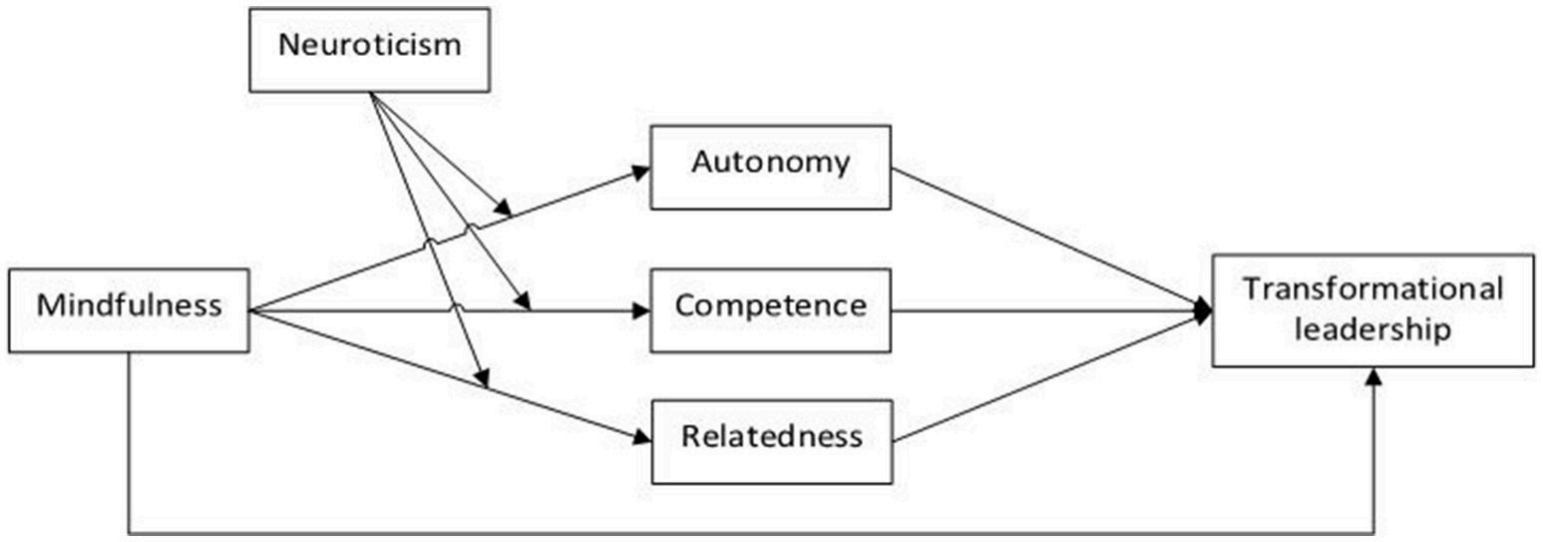
Theoretical model.

We further adopt a regulatory approach to propose that mindfulness also enhances adaptive cognitive functioning through influencing rumination or catastrophizing and general negative emotional reactivity (Barnhofer et al., 2011; Prins et al., 2014). Put more simply: meta-analyses have shown that mindfulness is related to reduced distress among working adults (e.g., Virgili, 2015) and to enhanced emotion regulation (Mesmer-Magnus et al., 2017). Brain research on the effects of mindfulness on the amygdala and prefrontal control-mechanisms are in line with this finding (Tang et al., 2015). Research has also shown that mindfulness and neuroticism interact to produce effects on emotional reactivity and mood: when mindfulness is high, effects of high neuroticism or catastrophizing can be neutralized (Hülsheger et al., 2013; Prins et al., 2014). Moreover, especially the ability to describe inner experiences seems to be helpful in dealing with negative emotions (Barnhofer et al., 2011). This is important for leaders as well, since research has shown that leader stress influences leader behavior (Harms et al., 2017). Negative aspects that seem to threaten leaders' well-being and consequently diminish their transformational leader behaviors are mostly depression, anxiety and workplace alcohol consumptions (Byrne et al., 2014). Therefore, mindfulness as a tool to help cope with these situations and emotions may be fruitful. In this sense, mindfulness may specifically help leaders to display transformational leadership when neuroticism (of negative emotion and distress) is high. In contrast, when leaders are already emotionally stable and less prone to behaving overrun by negative affect in stressful situations, mindfulness may be less influential for fostering transformational leadership. Therefore, we propose that neuroticism moderates the relationship between mindfulness and psychological need satisfaction. In sum, we argue that the relationship between leader mindfulness and transformational leadership may be moderated by emotional stability and mediated by psychological need satisfaction. Figure 1 depicts the theoretical research model.

Our research makes several theoretical contributions to the literature. First of all, we outline specifically how mindfulness may benefit transformational leadership, and hereby build on and extend previous theorizing on the specific link between mindfulness and transformational leadership (Pinck and Sonnentag, 2017). By studying mindfulness in relationship with transformational leadership, this study supports the emerging field on leader mindfulness (Reb and Atkins, 2015). Second, by adopting a perspective based on self-determination theory, we advance the research by identifying an underlying mechanism that can explain mindfulness' effects on transformational leadership. In the leadership literature, most research has focused on how transformational leadership leads to followers' psychological need satisfaction, rather than on psychological need satisfaction of leaders themselves (Trépanier et al., 2012), although it may be a vital process through which leaders are motivated for their crucial roles in organizations. Third, we advance the field on leader personality research through proposing that personality does not only directly influences leadership (Bono and Judge, 2004), but may also be a moderating factor influencing transformational leadership. More specifically, we propose that the negative effects of neuroticism may be mitigated by mindfulness. This addresses calls for research specifically for personal variables that moderate between leader mindfulness and transformational leadership (Pinck and Sonnentag, 2017). Fourth, we explore antecedents of transformational leadership that can be influenced by leaders themselves. From a practical standpoint, the relevance of mindfulness for transformational leadership indicates changing one's mindset or following a mindfulness training protocol may enhance transformational leadership on the work floor. This is valuable information for organizations who have already recruited leaders and want them to perform optimally.

### The Direct Relationship of Mindfulness and Transformational Leadership

We draw on conservation of resources theory to propose that mindfulness is positively related to transformational leadership. Conservation of resources theory proposes that people possess a finite number of resources, e.g., self-esteem, time, knowledge, or conditions (job security or social relationships at work). In an effort to prevent suffering, people strive to obtain and protect these resources (Hobfoll, 2001). Individuals who lack these resources will experience stress, and are prone to further loss. Therefore, depletion may lead individuals to adopt defensive postures to conserve whatever they have left (Hobfoll, 2001). Research has also shown that e.g., depleted employees score higher on burnout (Halbesleben, 2006). Since transformational leaders inspire their followers, intellectually stimulate them and are individually considerate (Bass, 1999), they extend more effort into their leadership role than e.g., transactional leaders. Performing transformational leader behavior therefore requires sufficient personal resources (Byrne et al., 2014). We propose that mindfulness supports the conservation of resources (Hobfoll, 2001), since it enhances self-regulation and therefore self-care (Brown and Ryan, 2003). In this sense, mindfulness will help protect leaders from a resource deficit and a negative stress-cycle with further depletion. A recent meta-analysis shows that mindfulness is negatively related to both stress and burnout (Mesmer-Magnus et al., 2017). Therefore, we propose that when trait mindfulness is a (personal) resource for leaders, it is more likely they enact transformational leadership.

Mindfulness as a personal resource may benefit several leadership styles, not only transformational leadership. Nevertheless, considering the relevance of transformational leadership in today's VUCA world (Rodriguez and Rodriguez, 2015), we are specifically interested in how mindfulness impacts transformational leadership. In addition, based on theorizing and empirical research with regards to mindfulness, we proposed that mindfulness has distinct effects on the four dimensions of transformational leadership specifically.

Mindfulness could enhance (1) idealized influence since it is related to authentic functioning and work engagement (Leroy et al., 2013). The enhanced authentic functioning may help display the idiosyncratic and personal leader charisma, while the resulting vigor and motivation from work engagement may also be inspirational for employees. Leaders who score high on mindfulness in general are seen as inspirational and influential role models within organizations because they can help solve difficult problems, make balanced decisions, are able to regulate their emotional responses to stressful events while being present with their employees (Bunting, 2016). Second, mindfulness should help the leaders' propensity to provide (2) intellectual stimulation, since it helps to see situations with a “beginners' mind” (Brown and Ryan, 2003; Dane, 2011), allows leaders to observe situations more objectively (Bishop et al., 2004; Pinck and Sonnentag, 2017) and helps overcome automatic processes and cognitive biases (Brown et al., 2007; Karelaia and Reb, 2015). Mindfulness also enhances flexibility, curiosity and therefore creativity (Langer and Moldoveanu, 2000; Weick and Putnam, 2006). In sum, this helps leaders to provide their employees with novel ideas and perspectives. Third, mindfulness should also be able to influence (3) inspirational motivation since it supports value-driven, ethical behavior (Ruedy and Schweitzer, 2010; Eisenbeiss and Van Knippenberg, 2015; Guillén and Fontrodona, 2018). That way, mindfulness helps leaders to better understand and therefore act in accordance with their values and goals (Brown and Ryan, 2003; Glomb et al., 2011; Pinck and Sonnentag, 2017). Mindfulness can thus help the leader to clarify values and be an authentic and engaged role model (Leroy et al., 2013), present and able to inspire and motivate employees. Last, mindfulness helps leaders (4) to consider each employee individually (i.e., individualized consideration) through enhanced awareness when communicating with employees (Bunting, 2016; Pinck and Sonnentag, 2017). This should help leaders to better regulate their (possible negative and automatic) reactions to employees, while also taking into account the exterior circumstances in which the employee operates (Dane, 2011). The awareness in the present moment should help leaders to “consider subordinates' personal needs and wishes before acting” (Pinck and Sonnentag, 2017, p. 2). Research has already shown that mindfulness enhances perspective taking and empathic concern in romantic couples (Block-Lerner et al., 2007). So there are several ways through which mindfulness supports transformational leadership.

In sum mindfulness enhances (1) idealized influence since it relates to authentic functioning, it supports (2) intellectual stimulation of employees because of the enhanced objectivity, overcoming of biases and enhanced creativity, it influences (3) inspirational motivation since it supports value-driven, ethical behavior and a better understanding of own values and needs and mindfulness can also be related to (4) individualized consideration since the enhanced awareness when communicating with employees combined with the increased empathy and decreased emotional reactivity, should enable the leader to make more idiosyncratically helpful and supportive decisions with regards to employees. Therefore, we propose hypothesis 1:

Hypothesis 1: Mindfulness is positively related to transformational leadership

### The Mediating Role of Psychological Need Satisfaction

We draw on self-determination theory (SDT, Deci and Ryan, 2008), to advocate that psychological need satisfaction may be one of the underlying processes that help explain how leader mindfulness impacts transformational leadership. Previous research has already established the importance of psychological need satisfaction for transformational leaders (Trépanier et al., 2012). In this study we build on that research by proposing that mindfulness may positively impact a leaders' self-determination. More specifically, mindfulness can be specifically associated with enhancing autonomy, competence and relatedness need satisfaction. Autonomy refers to a sense of volition and freedom (Van Den Broeck et al., 2010). Mindfulness influences autonomy since it is related to leader self-mastery and self-regulation (King and Haar, 2017). Mindfulness is also characterized by an open, present moment attention span in which information can be processed more accurately (Karelaia and Reb, 2015). This way, mindfulness enhances creativity (Carson and Langer, 2006; Zheng and Liu, 2017) and decreases decision biases in decision making (Kiken and Shook, 2011; Hafenbrack et al., 2014). Furthermore, mindfulness helps to create a “space” between trigger and response, in which difficult situations can be adequately assessed and emotional responses can be better managed. This is also shown by neuroscience research indicating that mindfulness helps regulate affect through enhancing prefrontal cortex inhibition of the amygdala (emotional) responses (Goleman and Davidson, 2017). All of this may contribute to a feeling of volition. Furthermore, when leaders' autonomy needs are met, they are more likely to be able to stimulate employee autonomy, which is a part of transformational leadership (Bass, 1999). In addition, SDT posits that autonomy deficits are sought to be replenished (Deci and Ryan, 2008). Therefore, based on a resource-maintenance perspective on autonomy (Van Quaquebeke and Felps, 2016), a leader who is low on autonomy is less likely to be able to provide autonomy to employees. Conversely, autonomy need satisfaction will lead to more transformational leadership.

*Hypothesis 2a: Leader autonomy need satisfaction mediates the relationship between mindfulness and transformational leadership*.

Competence, the second psychological need, can be described as succeeding at challenging tasks (Van Den Broeck et al., 2010). Mindfulness enhances competence since it is directly related to self-rated job performance (Mesmer-Magnus et al., 2017), as well as a higher leader effectivity rated by the employees (Waldron and Ebbeck, 2015; Wasylkiw et al., 2016) and the leaders' managers (King and Haar, 2017). Mindfulness leads to performance and effectivity through several possible pathways: the effect of mindfulness on (emotional) self-regulation, information processing and decision making will certainly contribute to leader effectivity and consequently the feeling of competence (Karelaia and Reb, 2015). In addition, the positive association of mindfulness with efficacy or confidence, work effort and job satisfaction, as well as the negative relationship with stress, burn-out and work withdrawal (Mesmer-Magnus et al., 2017) are also relevant in this context. Moreover, through the process of distancing and (re)perceiving a certain situation, the leader can become less controlled by thoughts and emotions (Pircher Verdorfer, 2016), enhancing a feeling of personal competence as well. When leaders feel competent and effective, when they “do the right thing,” they act as role models (Kelloway and Barling, 2000). This provides inspirational motivation for employees, which is a part of transformational leadership. In addition, high levels of leader self-esteem, which can be based on competence, are also associated with transformational leadership (Gretchen and Robert, 1996). Therefore, we propose the following hypothesis:

*Hypothesis 2b: Leader competence need satisfaction mediates the relationship between mindfulness and transformational leadership*.

The last psychological need, relatedness, refers to being connected to others and feeling cared for (Van Den Broeck et al., 2010). First of all, mindfulness has been shown to influence employee relatedness need satisfaction (Reb et al., 2014). Since a work relationship is a two-way street (Bauer and Green, 1996), the reverse may also be true: when the leader-employee relationship is good, it is plausible that the relatedness need satisfaction of the leader is also more satisfied. Furthermore, the association between mindfulness and interpersonal relations at work has also been shown in a recent meta-analysis (Mesmer-Magnus et al., 2017). Mindfulness may help leaders to connect with their employees through being present in the moment and through listening attentively (Ucok, 2006). It helps leaders to communicate more clearly and develop trust (Kearney et al., 2013; Frizzell et al., 2016; Roberts and Williams, 2017), which also leads to good working relationships. This is in line with research showing that mindfulness increases empathy (Block-Lerner et al., 2007). When leader relatedness need satisfaction is high, and the connection with their employees is satisfactory, the leader is able to pay more attention to developmental needs of followers. This strengthens the supporting and coaching role of leaders, which relates to the individualized consideration dimension of transformational leadership (Bass, 1999).

*Hypothesis 2c: Leader relatedness need satisfaction mediates the relationship between mindfulness and transformational leadership*.

### The Moderating Role of Neuroticism

First of all, mindfulness relates to an enhanced attention and awareness in the present moment, without judgment. Generally speaking, it is a broad and open awareness (Dane, 2011). The level of mindfulness can vary from person to person, but is it also trainable (Brown and Ryan, 2003). A very prominent feature or working mechanism of mindfulness is the enhanced self-regulation, which is also captured in a very early definition from Kabat-Zinn (1982, p. 34): “Meditation can be defined as the intentional self-regulation of attention from moment to moment.” Through the focus on the present moment, there is less distraction from worries about the future or past (Brown and Ryan, 2003). Through accepting the present moment as it is, stress is diminished (Shapiro et al., 2015) and negative effects from excessive worrying, e.g., catastrophizing, are counteracted (Prins et al., 2014). Mindfulness is thus a way to avoid automatic assumptions or (emotional) reactions. Research indeed indicates that mindfulness is negatively related to neuroticism and negative affect (Giluk, 2009). It enables leaders to respond more reflective instead of reactive, which is also shown by brain research on amygdala responsiveness to emotional stressors (Goleman and Davidson, 2017). Therefore, mindfulness is especially helpful to enhance emotion regulation and respond effectively to numerous stressors or encounters in the work place. Research has also shown that when mindfulness is high, potential negative effects of high neuroticism can be neutralized (Barnhofer et al., 2011). Based on these findings, we propose that mindfulness is especially effective when leader neuroticism is high.

Neuroticism can also be related to psychological need satisfaction. In earlier research that provided the basis for the Self-Determination Theory, it was posited that there are individual differences with regards to “causality orientations” that can be captured within autonomy, control and impersonal orientation. These causality orientations influence how people interpret and respond to events (Deci and Ryan, 1985). Interestingly, the authors posit that the causality-orientations have relationships with personality constructs. Specifically, they found associations between the impersonal causality orientation (which is equivalent to a feeling of a lack of competence) and negative emotions (i.e., neuroticism; Deci and Ryan, 1985). Other research e.g., reports positive associations between autonomy and emotional stability (Barrick and Mount, 1993). It is conceivable that neuroticism interacts with psychological need satisfaction in several ways. First, when negative affect is high, this may impede a feeling of competence. This is implicitly shown in meta-analytic research with regards to the effects of work demands on job performance: when stress and negative affect are high, because of e.g., work-life conflict or role ambiguity, performance suffers (Gilboa et al., 2008). Consequently, one may feel less competent. Second, emotional stability may influence autonomy need satisfaction: when one is emotionally stable, one is *internally free* from negative emotionality that may be distracting or impede work performance. Third, emotional stability or neuroticism can have an impact on the formation of work relationships: high neuroticism may e.g., impede one's capacity to be present for other people on the work floor and reduce the ability to connect to employees.

Bringing these perspectives together, we propose that neuroticism acts as a moderator in the relationship between mindfulness and psychological need satisfaction. More specifically, we propose that mindfulness-based emotion regulation will enhance autonomy need satisfaction through aiding the *personal freedom* from (emotional) reactivity. Second, it will aid competence need satisfaction through enhanced *leader effectivity*. And third, it will enhance relatedness need satisfaction through enhanced *attentive listening and communication* abilities (Kets de Vries, 2014; Bunting, 2016).

*Hypothesis 3a: Neuroticism moderates the relationship between mindfulness and autonomy need satisfaction, such that the relationship is stronger for those high in neuroticism*.
*Hypothesis 3b: Neuroticism moderates the relationship between mindfulness and competence need satisfaction, such that the relationship is stronger for those high in neuroticism*.
*Hypothesis 3c: Neuroticism moderates the relationship between mindfulness and relatedness need satisfaction, such that the relationship is stronger for those high in neuroticism*.

### The Moderated Mediation Model

Incorporating a regulatory approach into the self-determination perspective, our research proposes an integrated model in which neuroticism moderates the mediating mechanism of psychological need satisfaction in the relationship between mindfulness and transformational leadership. Employees with high levels of neuroticism will see the most benefits with regards to the effect of mindfulness on psychological need satisfaction, since mindfulness will act as a buffer for neuroticism and make sure that leaders high in neuroticism can also be high on autonomy, competence and relatedness. Consequently, leaders high in need satisfaction will have more resources to engage in transformational leader behaviors. In constrast, leaders low in neuroticism might experience a lower self-regulatory impact of mindfulness on psychological need satisfaction. Mindfulness may still have a direct impact on psychological need satisfaction and transformational leadership, but we hypothesize the effect will be smaller. These proposed relationships are summarized in hypothesis 4.

*Hypothesis 4: Neuroticism moderates the mediating effect of psychological need satisfaction on leader mindfulness and transformational leadership, such that the mediating effect is stronger for those high rather than low on neuroticism*.

## Methods

### Sample and Procedure

The data from this study came from head nurses in leadership positions in elderly care homes. Structured paper-and-pencil questionnaires were administered in October-November 2017. The supervisors of the organizations were informed on the research goal and asked to identify head nurses who could participate during breaks. To enhance data quality, a paper-and-pencil data collection was issued on site. In total, 108 elderly homes in Belgium were visited, of which 277 head nurses filled out the questionnaire. The data were part of a larger survey. See Table 1 for the demographic information.

**Table 1:** Demographic information.

**Variable**	**277 supervisors**
Average age (SD)	45.38
Gender (% female)	78.3
Tenure as an employee (in years)	15.06
Tenure as a supervisor (in years)	11.27
Span of control	19.24
**EDUCATIONAL BACKGROUND**	
Vocational secondary education (%)	1.8
Technical secondary education (%)	1.4
General secondary education (%)	1.1
Higher education (%)	81.5
University education (%)	10.8

#### Sample

Fifty-five head nurses were male (19.9%) and 217 were female (78.3%), 5 head nurses did not report their gender. The average age was 45 years old (SD 9.7), ranging from 22 to 74 years. The average tenure as a nurse was 15 years (SD 9.2), ranging from 0 to 37 years. The average tenure as a head nurse was 11.3 years (SD 8), ranging from 0 to 35 years. The average span of control was 19.2 employees (SD 8.9), ranging from 2 to 50. Participants' educational background: ranged from 5 with vocational secondary education (1.8%), 4 with technical secondary education (1.4%), 3 with general secondary education (1.1%), 225 with higher education (81.2%) and 30 with university education (10.8%).

#### Common Method Bias

Several apriori procedures were included to minimize common-method variance (as proposed in Podsakoff et al., 2003). First, head nurses were ensured the data would be treated *confidentially*, without any repercussions for them personally. Second, we only used measures from which the items were carefully constructed and from which the *psychometric properties* were demonstrated in prior research. Third, we made sure there was *psychological separation* of the focal constructs in the survey, by dividing them into different survey “chapters.” This way, questionnaires were separated by themes, headings and a blank space under the page (each new questionnaire also started on a new page). Finally, statistical procedures were used to assess the potential level of common-method variance.

### Measures

In order to obtain high content validity, all measures were derived from literature. All items of the surveys below were administered in Dutch and rated on a 7-point scale (1 = I completely disagree to 7 = I completely agree). Head nurses rated their own levels of mindfulness, transformational leadership, need satisfaction and emotional stability. Self-report measures are appropriate for these variables, since especially the level of mindfulness, need satisfaction and emotional stability are “private events” that can best be assessed by the focal employee (Conway and Lance, 2010). In addition, the measurement of transformational leadership has a long-standing tradition of being measured with self-report questionnaires as well (see e.g., Kovjanic et al., 2013; Besieux et al., 2015; Bui et al., 2017). Leaders may be more aware of the subtle things they do to be a role model, provide inspiration, stimulate employees intellectually and consider their individuality. Moreover, the answers to the self-report questionnaire can be seen as behavioral intentions that represent a readiness to act as a transformational leader. Based on the theory of reasoned action (Fishbein and Azjen, 1975), intentions are good predictors of volitional behaviors. Therefore, self-report measures can also effectively measure leadership.

#### Transformational Leadership

Transformational leadership was measured using 12 items from the Multifaceted Leadership Questionnaire (MLQ; Bass and Avolio, 1990; Avolio et al., 1999). Because of time constraints, we chose a more concise measure, as was done in previous studies with busy leaders (see e.g., Peterson et al., 2012). The short MLQ version with 12 items concerning 4 dimensions has been validated and used in previous research (Tims et al., 2011; Bae et al., 2013; Song et al., 2013). Items were translated in Dutch using a translation-back translation procedure with two iterations (Brislin, 1990). In addition, the items were adapted to the context (i.e., nursing homes), therefore the general “others” in the scale was replaced by “my nursing staff.” The scale included 3 items for each of the four dimensions of transformational leadership, including idealized influence (e.g., “I make sure my nursing staff feels good when I am around.”), inspirational motivation (e.g., “I use a few simple words to express what we can do.”), intellectual stimulation (e.g., “I help my nursing staff to think in new ways about old problems.”) and individual consideration (e.g., “I help my nursing staff to develop themselves.”). An English translation of the exact items, as well as the factor loadings, can be found in the Appendix. In line with previous research and recommendations from authors, these items were combined into one factor (Cronbach's α = 0.899) (Bycio et al., 1995; Judge and Bono, 2000; Bono et al., 2007). This is because all the measured behaviors are expected to contribute to transformational leadership (Bass and Avolio, 1990; Avolio et al., 1999), which is also supported by the medium to high intercorrelations between the four dimensions (*r* = 0.51–0.80).

#### Need Satisfaction

Need satisfaction was measured with 18 items from the Work-Related Basic Need Satisfaction Scale (W-BNS). The scale included 6 items for each of the three dimensions, comprising the basic psychological needs for autonomy (e.g., “The tasks I have to do at work are in line with what I really want to do.”), competence (e.g., “I really master my tasks at my job.”) and relatedness (e.g., “At work, I feel part of a group.”). The scale has been extensively validated in Dutch (Van Den Broeck et al., 2010) and showed a good internal consistency (Cronbach's α = 0.894). The Cronbach's alpha was also examined for each factor separately: autonomy (α = 0.817), competence (α = 0.86), relatedness (α = 0.785). Since the three needs are conceptually different, we assessed their effects separately, as was done in previous research.

#### Mindfulness

Mindfulness was measured with 15 items from the Mindful Attention and Awareness Scale (Brown and Ryan, 2003), which assumes that mindfulness has a unidimensional structure. This questionnaire measures awareness and attention with regards to what is taking place in the present (e.g., “I could be experiencing some emotion and not be conscious of it until sometime later.”) and has been validated in Dutch (Schroevers and Nyklíček, 2008). The internal consistency in the present study was satisfactory (Cronbach's α = 0.857).

#### Neuroticism

Neuroticism was measured with two items for emotional stability from the short 10-item Big Five Inventory (BFI) questionnaire for research contexts with time constraints (Rammstedt and John, 2007): I see myself as someone who… (1) is relaxed, handles stress well; (2) gets nervous easily. The questionnaire was designed to capture as much variance as possible with the smallest item number as possible. Linked to this purpose, the two-item scale can present lower internal consistency (Rammstedt and John, 2007). On the other hand, the (test-retest) reliability and overall validity of this scale have been shown to be adequate and item loadings are similar to those of the larger Big Five Inventory with 44 items (Rammstedt and John, 2007). Convergent validity with the NEO-PI-R (and its facets) has also been established (Rammstedt and John, 2007). The scale has been successfully used in previous research (e.g., Daly et al., 2014). Research has shown that Cronbach's alpha underestimates true reliability of a two-item scale (Eisinga et al., 2013). Therefore the Spearman-Brown coefficient was calculated (Eisinga et al., 2013), which in this case showed similar results (Spearman-Brown = 0.591; Cronbach's alpha α = 0.590). The reliability results were in line with other studies using 10-item personality measures (e.g., Perreault et al., 2016). The correlation between the two items is probably not high enough to yield more than moderate reliability indices (Pearson's *r* = 0.42) (Eisinga et al., 2013). A lower reliability is to be expected, since it is a very small questionnaire designed to efficiently capture different variance in emotional stability (Rammstedt and John, 2007).

#### Demographic Control Variables

We controlled for supervisors' gender, age, tenure as an employee (nurse), tenure as a supervisor (head nurse) and span of control. Gender has been shown to be related to need satisfaction, with women scoring slightly higher (Van Den Broeck et al., 2008b). Furthermore, tenure and span of control were added, since we expect this to influence the three subsdimensions of need satisfaction of the supervisor (Bernerth and Aguinis, 2016). Tenure and span of control may influence the need for autonomy and competence, since a high tenure may ensure a supervisor to work more autonomous and a high span of control may increase the workload to diminish the amount of autonomy. Tenure and span of control may also influence the need for relatedness, since a high tenure may have caused friendships to develop, but a high span of control may limit the time a supervisor spends with his/her colleagues or employees.

### Analytical Strategy

First, to examine the factor structure of each construct separately, a regular factor analysis was performed in SPSS 24 to see of each of our concepts loaded significantly on the specified factors. Second, confirmatory factor analyses (CFA) were performed with Lavaan in R (Rosseel, 2012) to ensure the discriminant validity of the measures. Third, to test the mediation and moderation effect, multiple regression analyses were conducted to assess each component. The SPSS macro developed by Preacher et al. (2007) which uses a bootstrapping method, was then used to further estimate the bias-corrected confidence estimates for the mediation and moderation, as well as for testing the moderated mediation hypothesis. In this last step, the significance of the conditional indirect effects for different values of the moderator variable (– 1 SD and + 1 SD) are estimated as well. Bootstrapped confidence intervals are interesting to use since they avoid problems with non-normal distributions of indirect effects (MacKinnon et al., 2004). Prior to any analyses, the interaction variables were mean-centered (Cohen et al., 2003). To aid interpretation with the moderation Figure, the variables were not centered there.

## Results

### Preliminary Analyses

#### Common Method Bias Analyses

To examine the level of common method bias, Harman's (1976) recommendation was followed: all the variables in the study were loaded into an exploratory factor analyses. Eleven factors with eigenvalues >1 emerged. The first factor only accounted for 22.87% of the variance and the 11 factors accounted for 63.65% of the total variance. The first factor only accounts for a small portion of the variance. Furthermore, CFA (see below) indicated that a one factor solution for our measurement model showed very poor fit and differed significantly from the hypothesized model (Δ (*SB*)χ^2^(6) = 20.829, *p* < 0.001). Therefore, according to this first analysis, common method bias did not seem to be a serious threat to the validity of the analyses. Since this procedure in itself is not a perfect measure of common method bias (Podsakoff et al., 2003), we also conducted an analysis in which we added an unmeasured latent factor to the measurement model. If such a method factor existed, the model would have a better fit compared with the model without such a factor (Podsakoff et al., 2003). Adding this additional latent factor did result in a slightly better fit (χ^2^ = 1312.800, d.f. 880, p < 0.05), indicating the possibility of a small amount of common method bias influencing the results despite our apriori efforts to counteract it. This is unfortunate, but it does not exclude that there may be merit to our results (Conway and Lance, 2010), especially when interactions are concerned (Evans, 1985; Siemsen et al., 2010).

#### Factor Loadings

The regular factor structure of each construct was examined separately using principal axis factoring with a specified loading with a fixed number of factors (= 1). Principal axis factoring was used since it does not assume multivariate normality and thus provides a more robust test of the data (Yong and Pearce, 2013).

The factor loadings for mindfulness ranged from 0.347 to 0.708. Since 2 items loaded lower than 0.4 on the general factor, it was decided to omit them from further analysis (see Appendix). This slightly improved the Cronbach's alpha (from 0.857 to 0.861). The factor loadings for need satisfaction were analyzed separately for each of the three needs (see below). The factor loadings of autonomy need satisfaction ranged from 0.579 to 0.737. The factor loadings from competence need satisfaction ranged from 0.565 to 0.904. The factor loadings from relatedness need satisfaction ranged from 0.333 to 0.701. It was decided to omit the lowest loading variable (see Appendix). This increased Cronbach's alpha from 0.785 to 0.801. The factor loadings for transformational leadership ranged from 0.581 to 0.757. Factor loadings of emotional stability were not examined, since a factor with 2 variables is only reliable when the variables themselves are highly correlated (>0.70) (Yong and Pearce, 2013), which was not the case (*r* = 0.42).

#### Measurement Model

Confirmatory factor analyses (CFA) were performed to ensure the discriminant validity of the measures. There is a good fit between the hypothesized model and the data when χ ^2^/df is lower than 3, the Comparative Fit Index (CFI) and Tucker-Lewis Index (TLI) are close to 0.95, the root mean square error of approximation (RMSEA) and the standardized root mean square residual (SRMR) are close to 0.06 and 0.08, respectively (Hu and Bentler, 1999). To adjust for non-normality with ordinal data, the Satorra-Bentler (*SB*)χ^2^ difference test was used. Results are summarized in Table 2.

**Table 2:** Model fit.

**Models**	**(*SB*)χ^2^**	**df**	**χ ^**2**^/df**	**Δ (*SB*)χ^2^**	**Δ df**	**CFI**	**TLI**	**RMSEA**	**SRMR**
Six factor-model: The hypothesized model	1442.030	887	1.63			0.843	0.833	0.056	0.068
Four factor-model: Need satisfaction clustered	1463.955	893	1.64	20.829^**^	6	0.838	0.829	0.057	0.072
Three factor-model: Mindfulness + need satisfaction	2199.239	899	2.47	303.3^***^	12	0.624	0.604	0.068	0.092
Three factor-model: Need satisfaction + leadership	2268.584	899	2.52	342.08^***^	12	0.605	0.584	0.088	0.098
Three factor-model: Leadership + neuroticism	1865.867	899	2.07	264.11^***^	12	0.724	0.710	0.074	0.081
Two factor-model Mindfulness + need satisfaction	2247.121	901	2.49	344.33^***^	14	0.610	0.591	0.088	0.097
Two factor-model: Need satisfaction + leadership	2328.068	901	2.58	375.44^***^	14	0.587	0.566	0.090	0.105
One factor-model: All variables combined	2736.812	902	3.03	433.65^***^	15	0.462	0.436	0.103	0.114

*(SB)χ^2^, Satorra-Bentler adjusted chi square coefficient; df, degrees of freedom; CFI, comparative fit index; TLI, Tucker-Lewis index; RMSEA, root mean square error of approximation; SRMR, standardized root mean square of approximation. Both Δ χ^2^ and Δ df were based on the comparison with the six-factor model*.

** , p < 0.001;

*** *, p < 0*.

The confirmatory factor analysis with six factors (transformational leadership, emotional stability, mindfulness and three for autonomy, competence and relatedness need satisfaction) had the best fit, albeit somewhat below standard recommendations [(*SB*)χ^2^ (887) = 1442.030, χ ^2^/df = 1.63, CFI = 0.84, TLI = 0.83, RMSEA = 0.056, SRMR = 0.068]. This is not necessarily problematic, since the model fit indices may be sensitive to a large number of parameters (e.g., data structure, the particular index and sample size) and golden standards for model fit may not have a huge utility (Marsh et al., 2009). In addition, scholars have argued the importance of considering theory-relevant criteria for assessing model fit (Barrett, 2007). Taking this into account, our hypothesized 6 factor-model fit was significantly better than a four factor-model with the need satisfaction scales clustered [Δ χ^2^ (893) = 20.829^***^], a three factor-model with mindfulness and need satisfaction [Δ χ^2^ (899) = 303.3^***^], a three factor-model with need satisfaction and leadership [Δ χ^2^ (899) = 342.08^***^], a three factor-model with leadership and neuroticism [Δ χ^2^ (899) = 264.11^***^], a two factor-model with mindfulness and need satisfaction [Δ χ^2^ (901) = 344.33^***^], a two factor-model with need satisfaction and leadership [Δ χ^2^ (901) = 375.44^***^] and a one factor-model [Δ χ^2^ (902) = 433.65^***^]. In sum, the CFA shows that all items loaded on their hypothesized factors. The three needs from basic need satisfaction (competence, autonomy, and relatedness) could indeed be further examined separately.

#### Intercorrelations of Study Variables

The means, standard deviations, and correlations are presented in Table 3. The correlations show that mindfulness is significantly negatively associated with neuroticism (*r* = −0.325, *p* < 0.01). Furthermore, mindfulness is positively associated with need satisfaction: autonomy (*r* = 0.393, *p* < 0.01), competence (*r* = 0.431, *p* < 0.01) and relatedness need satisfaction (*r* = 0.347, *p* < 0.01). Lastly, mindfulness and transformational leadership are positively associated (*r* = 0.0.248, *p* < 0.01). Neuroticism is also negatively associated with need satisfaction (autonomy *r* = −0.309, *p* < 0.01; competence: *r* = −0.432, *p* < 0.01), relatedness: *r* = −0.201, *p* < 0.01) and transformational leadership (*r* = −0.377, *p* < 0.01). Next, transformational leadership is significantly associated with autonomy (*r* = 0.288, *p* < 0.01), competence (*r* = 0.357, *p* < 0.01) and relatedness need satisfaction (*r* = 0.303, *p* < 0.01).

**Table 3:** Descriptive statistics and intercorrelations.

		**Mean**	**SD**	**1**	**2**	**3**	**4**	**5**	**6**	**7**	**8**	**9**	**10**
1	Gender^a^	0.8	0.40										
2	Age	45.38	9.69	0.017									
3	Tenure^1^	15.06	9.19	0.089	0.501^**^								
4	Tenure^2^	11.27	8.04	−0.103	0.610^**^	−0.049							
5	Spoc	19.24	8.88	−0.014	0.075	0.120	0.063						
6	Mindfulness	5.15	0.81	0.040	0.177^**^	0.026	0.156^*^	−0.41					
7	Neuroticism	2.85	1.04	0.110	−0.102	0.022	−0.077	−0.082	−0.325^**^				
8	Autonomy	5.31	0.86	0.004	0.004	−0.157^*^	0.101	−0.069	0.393^**^	−0.309^**^			
9	Competence	5.74	0.76	−0.04	0.244^*^	0.080	0.233^*^	−0.030	0.431^**^	−0.432^**^	0.514^**^		
10	Relatedness	5.79	0.90	0.084	0.094	−0.023	0.129^*^	−0.151^*^	0.347^**^	−0.201^**^	0.513^**^	0.339^**^	
11	Transformational	5.52	0.56	0.089	0.127^*^	0.146^*^	0.41	−0.067	0.248^**^	−0.377^**^	0.288^**^	0.357^**^	0.303^**^

* *p < 0.05*,

** p < 0.01;

a Coded 0, male; 1, female;

1 Tenure as employee;

2 *Tenure as a supervisor; Spoc, span of control; N = 277*.

Furthermore, not all control variables were significantly associated with our core research variables (see Table 3). Age was significantly associated with mindfulness (*r* = 0.177, *p* < 0.01), competence need satisfaction (*r* = 0.244, *p* < 0.05) and transformational leadership (*r* = 0.127, *p* < 0.05). Tenure as a nurse was significantly associated with autonomy (*r* = −0.157, *p* < 0.5) and transformational leadership (*r* = 0.146, *p* < 0.05). Tenure as a head nurse was significantly associated with mindfulness (*r* = 0.156, *p* < 0.05), competence need satisfaction (*r* = 0.233, *p* < 0.05) and relatedness need satisfaction (*r* = 0.129, *p* < 0.05). Span of control was only significantly associated with relatedness need satisfaction (−0.151, *p* < 0.05). Following Becker's (2005) recommendations, only significant control variables were included in the relevant analyses. Furthermore, since age and tenure are highly correlated (*r* = 0.50, *p* < 0.01 with tenure as an employee; *r* = 0.61, *p* < 0.01), it was opted to control for tenure alone.

### Test of the Main Effect

Hypothesis 1 predicts that mindfulness is positively related to transformational leadership. We performed a linear regression and controlled for both tenure as a leader and span of control. The analysis shows that mindfulness is indeed positively associated with transformational leadership [*B* = 0.17, *p* < 0.001; *F*_(3)_ = 5.54, *p* = 0.001, *r*2 = 0.06]. Therefore, hypothesis 1 was supported.

### Test of the Mediation Effect

Hypothesis 2a proposes that autonomy need satisfaction mediates the relationship between mindfulness and transformational leadership. The results in Table 4 show that autonomy need satisfaction was positively associated with transformational leadership (*B* = 0.14, *SE* = 0.04, *p* < 0.01), after controlling for the effect of mindfulness. Furthermore, we examined the robustness of this effect by estimating bootstrapped confidence intervals for this mediation effect with 5,000 samples. The results show that the indirect effect of mindfulness on transformational leadership via autonomy need satisfaction was significant (*B* = 0.05, *SE* = 0.02, CI = [0.02, 0.10]). Therefore, hypothesis 2a was supported. Hypothesis 2b proposes that competence need satisfaction mediates the relationship between mindfulness and transformational leadership. The results in Table 4 show that competence need satisfaction was positively associated with transformational leadership (*B* = 0.22, *SE* = 0.05, *p* < 0.001), after controlling for the effect of mindfulness. Moreover, the effect of mindfulness became non-significant (*B* = 0.08, *SE* = 0.05, *p* > 0.05), indicating a full mediation. Furthermore, we examined the robustness of this effect by estimating bootstrapped confidence intervals for this mediation effect with 5,000 samples. The results show that the indirect effect of mindfulness on transformational leadership via competence need satisfaction was significant (*B* = 0.08, *SE* = 0.02, *p* < 0.001, CI = [0.04, 0.13]). Therefore, hypothesis 2b was supported. Hypothesis 2c proposes that relatedness need satisfaction mediates the relationship between mindfulness and transformational leadership. The results in Table 4 show that relatedness need satisfaction was positively associated with transformational leadership (*B* = 0.14, *SE* = 0.04, *p* < 0.001), after controlling for the effect of mindfulness. Furthermore, we examined the robustness of this effect by estimating bootstrapped confidence intervals for this mediation effect with 5,000 samples. The results show that the indirect effect of mindfulness on transformational leadership via relatedness need satisfaction was significant (*B* = 0.05, *SE* = 0.02, *p* < 0.001, CI = [0.02, 0.09]). Therefore, hypothesis 2c was supported.

**Table 4:** Moderation and mediation effects.

**Variables**	**Need satisfaction**			**Transformational leadership**		
	**M1**	**M2**	**M3**	**M4**	**M5**	**M6**
	**B(SE)**	**B(SE)**	**B(SE)**	**B(SE)**	**B(SE)**	**B(SE)**
**CONTROLS (STEP 1)**						
Tenure^1^	<0.01(0.01)	0.02(0.01)^**^	0.02(0.01)	0.00(0.00)	0.00(0.00)	0.00(0.00)
Spoc	−0.01(0.01)	−0.00(0.01)	−0.02(0.01)^*^	−0.00(0.00)	−0.00(0.00)	−0.00(0.00)
*R2*	0.02	0.06	0.04	0.01	0.01	0.01
*Δ R2*	0.02	0.06	0.04	0.01	0.01	0.01
*F for Δ R2*	1.90	7.71^**^	5.41^**^	0.82	0.82	0.82
*F*	1.90	7.71^**^	5.41^**^	0.82	0.82	0.82
**PREDICTORS (STEP 2)**						
Mindfulness	0.36(0.07)^***^	0.29(0.06)^***^	0.33(0.07)^***^	0.17(0.04)^***^	0.017(0.04)^***^	0.17(0.04)^***^
Neuroticism	−0.14(0.05)^**^	−0.22(0.04)^***^	−0.08(0.06)		
*R2*	0.19	0.30	0.15	0.07	0.07	0.07
*Δ R2*	0.17	0.24	0.11	0.06	0.06	0.06
*F for Δ R2*	24.47^***^	39.31^***^	14.77^***^	14.90^***^	14.90^***^	14.90^***^
*F*	13.38^***^	24.75^***^	10.40^***^	5.54^**^	5.54^**^	5.54^**^
Mfn^*^NEUROTICSM (step 3)						
	0.03(0.05)	0.05(0.04)	0.12(0.06)^*^			
*R2*	0.19	0.30	0.17			
*Δ R2*	0.00	0.003	0.02			
*F for Δ R2*	0.14	0.94	4.90^*^			
*F*	10.69^***^	19.98^***^	9.44^***^			
**MEDIATION (STEP 3)**						
Autonomy				0.14(0.04)^***^		
Competence					0.22(0.05)^***^	
Relatedness						0.17(0.04)^***^
*R2*				0.10	0.14	0.12
*Δ R2*				0.04	0.07	0.06
*F for Δ R2*				10.29^**^	19.75^***^	15.54^***^
*F*				6.89^***^	9.42^***^	8.30^***^

*N = 277*;

1 Tenure as a leader; Spoc, Span of control; M1/M4, autonomy; M2/M5, competence; M3/M6, relatedness;

* *p < 0.05*,

** *p < 0.01*,

*** *p < 0.001; Unstandardized coefficients were presented*.

### Test of the Moderation Effect

Table 4 presents the results from the moderation and mediation linear regression analyses. M1, M2, and M3 represent the moderation analysis for autonomy, competence and relatedness need satisfaction, respectively. Only the final regression model (including the interaction step) was included for reasons of parsimony. Furthermore, the models depicted only include control variables that explained variance (Hox, 2010), i.e., tenure as a leader (explained 8.3% variance in competence need satisfaction) and span of control (explained 2.8% variance in need for relatedness).

Hypothesis 3a proposes that neuroticism moderates the relationship between mindfulness and autonomy need satisfaction, such that the positive relationship of mindfulness and need satisfaction is higher at high levels of neuroticism (or low levels of emotional stability). As shown in Table 4 (see M1), the interaction term of mindfulness and neuroticism was not significantly related to autonomy need satisfaction (*B* = 0.02, *SE* = 0.05, *p* > 0.05). Therefore, hypothesis 3a was not supported.

Hypothesis 3b proposes that neuroticism moderates the relationship between mindfulness and competence need satisfaction, such that the positive relationship of mindfulness and need satisfaction is higher at high levels of neuroticism (or low levels of emotional stability). As shown in Table 4 (see M2), the interaction term of mindfulness and neuroticism was not significantly related to competence need satisfaction (*B* = 0.04, *SE* = 0.04, *p* > 0.05). Therefore, hypothesis 3b was not supported.

**Figure 2:**
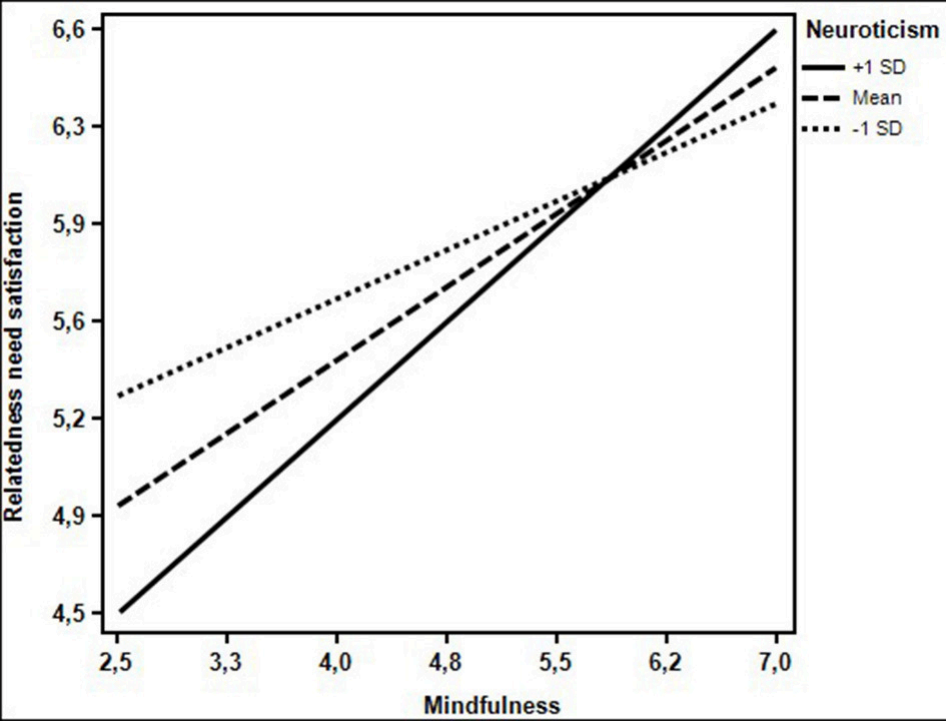
The moderating effect of neuroticism on the relationship between mindfulness and relatedness need satisfaction.

Hypothesis 3c proposes that neuroticism moderates the relationship between mindfulness and relatedness need satisfaction, such that the positive relationship of mindfulness and need satisfaction is higher at high levels of neuroticism (or low levels of emotional stability). As shown in Table 4 (see M3), the interaction term of mindfulness and neuroticism was significantly related to autonomy need satisfaction (*B* = 0.11, *SE* = 0.05, *p* < 0.05). With the interaction effect, 14.6% of variance in need satisfaction was explained. The interaction effect is visualized in Figure 2. To assess the robustness of this relationship, we assessed the same effect through a bootstrap procedure. Based on 5,000 bootstrapped samples, the same effect was found. When the level of neuroticism was low (1 SD below the mean), the relationship between mindfulness and relatedness need satisfaction was significantly positive (*B* = 0.22, *SE* = 0.09, *p* < 0.05, CI = [0.04, 0.40]). When the level of neuroticism was high (1 SD above the mean), the relationship between mindfulness and relatedness need satisfaction was significantly positive and stronger (*B* = 0.44, *SE* = 0.09, *p* < 0.001, CI = [0.26, 0.62]). This indicates that neuroticism actively influences the effect of mindfulness on need satisfaction, such that high neuroticism leads to a higher effect of mindfulness on need satisfaction. Or conversely, low emotional stability leads to a higher effect of mindfulness on relatedness need satisfaction. Therefore, hypothesis 3c was supported.

### Test of the Moderated Mediation Model

Hypothesis 4 stipulates a moderated mediation model, in which neuroticism moderates the mediating effect of need satisfaction on the relationship between mindfulness and transformational leadership. Since the moderating effect of neuroticism on autonomy need satisfaction and competence need satisfaction was not significant, the hypothesis can only be investigated for relatedness need satisfaction.

The conditional indirect effect was tested at three values of neuroticism: one standard deviation above the mean, the mean, and one standard deviation below the mean. The index of the moderated mediation in total was significant (*B* = 0.02, *SE* = 0.01, 95% CI = [0.001, 0.05]), indicating the existence of a moderated mediation effect. The results are presented in Table 5. This shows that at low levels of neuroticism, the conditional indirect effect is not significant (*B* = 0.03, *SE* = 0.02, 95% CI = [−0.005, 0.07]), since the confidence interval includes zero, whereas for mean neuroticism (*B* = 0.05, *SE* = 0.02, 95% CI = [0.02, 0.07]) and high neuroticism (*B* = 0.06, *SE* = 0.02, 95% CI = [0.02, 0.12]), the conditional indirect effect is significant. These results show that the indirect effect of mindfulness on transformational leadership through relatedness need satisfaction was observed only when neuroticism was medium to high. Conversely, the indirect effect of mindfulness on transformational leadership through relatedness need satisfaction was only observed when emotional stability was medium to low; indicating that mindfulness and neuroticism/emotional stability interact to create the effects through need satisfaction on transformational leadership. When neuroticism is low (or conversely emotional stability is high), there is no conditional indirect effect on transformational leadership. Together, these results support hypothesis 4 with regards to relatedness need satisfaction.

**Table 5:** Results of the conditional indirect effects.

**Conditions**	**Mindfulness (X) ->** **Relatedness need satisfaction (M) ->** **Transformational leadership (Y)**		
	**Indirect effect Ustd boot (boot SE)**	**[95% confidence interval]**	
Low neuroticism−1 SD (−1.03)	0.03 (0.02)	−0.005	0.07
Mean neuroticism(0.00)	0.05(0.02)^***^	0.02	0.08
High neuroticism+1 SD (1.03)	0.06(0.02)^***^	0.02	0.12

N = 277; Neuroticism was mean-centered beforehand; Ustd boot, unstandardized regression coefficient; Tenure as a leader and span of control as covariates. Bootstrap sample size, 5000; * p < 0.05 ** p < 0.01,

*** *p < 0.001*.

## Discussion

Our study investigated the antecedents of transformational leadership, with a focus on mindfulness. Previous research mostly studied the benefits of mindfulness for employees in organizations (Reb and Atkins, 2015), whereas the present study zooms in on the benefits of mindfulness for leaders themselves. Specifically, we focused on *if* and *when* mindfulness influences transformational leadership. Mindfulness was hypothesized to be specifically beneficial for transformational leaders in several ways: it may enhance idealized influence since is related to authentic functioning (Leroy et al., 2013), it may support intellectual stimulation based on the enhanced objectivity and creativity (Langer and Moldoveanu, 2000; Brown and Ryan, 2003; Bishop et al., 2004; Weick and Putnam, 2006; Dane, 2011; Karelaia and Reb, 2015), mindfulness may influence inspirational motivation since it supports value-driven, ethical behavior and a better understanding of own values and needs (Brown and Ryan, 2003; Ruedy and Schweitzer, 2010; Eisenbeiss and Van Knippenberg, 2015; Guillén and Fontrodona, 2018) and it may increase individualized consideration based on the enhanced awareness when communicating with employees, increased empathy and decreased emotional reactivity (Block-Lerner et al., 2007; Bunting, 2016; Pinck and Sonnentag, 2017). Besides theorizing based on mindfulness research, self-determination theory was our primary theoretical lens: we hypothesized that mindfulness also influences transformational leadership through aiding the satisfaction of the need for autonomy, competence and relatedness. Mindfulness can be related to (1) enhanced autonomy because it augments leader self-mastery and self-regulation (King and Haar, 2017). It can be related to (2) competence, since research has shown that it is directly related to leader job performance (Waldron and Ebbeck, 2015; Wasylkiw et al., 2016; Mesmer-Magnus et al., 2017) and mindfulness can be associated with increases in (3) the need for relatedness because it enhances empathy (Block-Lerner et al., 2007) and attentive listening (Ucok, 2006), which are core components of developing good relationships. Furthermore, we acknowledged that emotional distress may be a threat for effectively functioning as a transformational leader (Byrne et al., 2014; Harms et al., 2017). Therefore, we adopted a regulatory approach in which we investigated neuroticism as a moderator with regards to the effect of mindfulness on psychological need satisfaction and consequently transformational leadership. We proposed that mindfulness may exert its effects as an emotion regulatory mechanism, i.e., interacting with neuroticism to dampen the negative effects of high neuroticism (and emotional stress) on transformational leaders' performance. This is in line with previous research showing the importance of mindfulness for mitigating the negative effects of neuroticism and catastrophizing (Barnhofer et al., 2011; Prins et al., 2014), as well as brain research showing that mindfulness has an impact on the amygdala and prefrontal cortex which seems to indicate enhanced emotion regulation as well (Tang et al., 2015; Goleman and Davidson, 2017).

First of all, the analyses confirm that mindfulness is indeed related to transformational leadership, which is in line with a previous study (Pinck and Sonnentag, 2017). We add to this previous work by finding that this relationship is partially explained through the mediation of psychological need satisfaction, indicating that mindfulness influences the feeling of autonomy, competence and relatedness of leaders. Competence need satisfaction was a full mediator in this respect, while autonomy and relatedness were partial mediators. Through these findings we expand the knowledge in the emerging field of leader mindfulness (Reb and Atkins, 2015).

Second, we show that neuroticism interacts with mindfulness with regards to relatedness need satisfaction in such a way that when neuroticism was high, mindfulness had the highest impact on relatedness need satisfaction. Or conversely, when neuroticism was low, mindfulness had a smaller (but still positive) impact on relatedness need satisfaction (see Figure 2). This is in line with the idea that part of mindfulness' influence on relatedness need satisfaction exerts itself through an enhanced emotion regulation (Barnhofer et al., 2011; Prins et al., 2014; Tang et al., 2015; Goleman and Davidson, 2017). This was predicted by our inclusion of a regulation approach, in which we stated that mindfulness may enhance emotional regulation, and influence neuroticism, by focusing on the present moment and observing emotions rather than acting reactionary. The relationship with relatedness need satisfaction indicates that high neuroticism (combined with low mindfulness) might intervene with developing solid work relationships, perhaps because a highly neurotic leader may scare off or overburden employees. When a leader scores high on mindfulness, that effect can be mitigated and even lead to a better development of work relationships. Perhaps this is possible because a leader with higher scores on neuroticism is better equipped to understand emotional reactions and may be more able to be empathic through his/her own experience with emotional reactivity. The combination of neuroticism (emotionality) and mindfulness (positive coping), may then be a good example for (neurotic) employees and lead to better work relationships.

Third, our results indicate that the moderated mediation model for relatedness need satisfaction is also significant. This combines the effects explicated above and indicates that neuroticism is a boundary condition for the indirect effect of mindfulness on transformational leadership through relatedness need satisfaction. Within this moderated mediation model, the bootstrapped confidence intervals for one standard deviation above and below the mean also indicated that when neuroticism was low (or conversely when emotional stability was high), there was no conditional indirect effect any more on transformational leadership. This provides additional evidence for our regulatory hypothesis: when emotional stability is high, there is no longer an interaction effect with neuroticism on relatedness need satisfaction influencing transformational leadership. In this respect, the emotion regulation aspect of mindfulness no longer yield results, since there is no negative affect (neuroticism) to regulate any more. The regulatory effect of mindfulness may thus be one of the mechanisms in which mindfulness influences psychological need satisfaction, and relatedness need satisfaction specifically. Of course, emotion regulation was not measured *an sich*, but the specific patterns of interactions between mindfulness and neuroticism are in line with research positing that mindfulness works primarily through emotion regulation mechanisms (Feldman et al., 2007; Hülsheger et al., 2013; Tang et al., 2015).

### Theoretical Implications

Our research makes several contributions to the literature. First of all, we contribute to the literature on transformational leadership by focusing on antecedents rather than outcomes. Within this realm we focused specifically on cognitive/psychological rather than environmental/organizational antecedents of transformational leadership. In addition, the field of mindfulness research in organizational settings has just begun to explore mindful leadership (Reb and Atkins, 2015). Our study is one of the first studies to contribute to this field with regards to the relationship between mindfulness and transformational leadership, and the first study within this emerging field that only focuses on leader-central variables, rather than employee-related outcomes (see Pinck and Sonnentag, 2017). This is important, because expanding the knowledge on the interplay between mindfulness and transformational leadership may help us understand how transformational leaders can perform well in our changing environment (Rodriguez and Rodriguez, 2015). Furthermore, previous research focused on the vision, values and behaviors of transformational leaders (Sauer and Kohls, 2011), within this study we focus on the actual mindset of leaders, and the way in which they pay attention. Mindfulness was also proposed to be a personal resource for transformational leaders, and specifically related to transformational leader behaviors. We coupled this with a theoretical perspective based on self-determination theory and the importance of autonomy, competence and relatedness need satisfaction for leaders. Therefore, we contributed to the emerging field of leader mindfulness (Reb and Atkins, 2015), as well as on applications of the self-determination theory (Van Den Broeck et al., 2008a), by integrating a framework concerning leader cognition, mindfulness as a resource for transformational leader behavior and leader psychological need satisfaction for the emergence of transformational leadership. Moreover, through adopting a regulatory focus, we also examined whether or not mindfulness exerts its influence primarily through an interaction with neuroticism. Therefore, we add to the (theoretical) literature on mindfulness and emotion regulation (Hülsheger et al., 2013). This also addresses calls for research on the issue of personal variables that moderate between leader mindfulness and transformational leadership (Pinck and Sonnentag, 2017).

### Practical Implications

Our study has several practical implications. Firs, since leaders are role models within the organization, they can have a large impact on employees. Therefore, the positive effect of leader mindfulness can be expected to trickle down through the organization, in part because the leader will be more able to be transformational. When mindfulness infuses leadership, the leader will thus be more characterized by idealized influence, inspirational motivation, intellectual stimulation and individual consideration and therefore help employees to envision a joint, organizational goal to pursue.

Second, our results show that psychological need satisfaction is a mediating mechanism between mindfulness and transformational leadership. This indicates that mindfulness can increase the leaders' freedom to act (autonomy), effectivity (competence), and enhances work relationships (relatedness). The satisfaction of these three needs leads to a higher autonomous motivation, and likely to more energy to poor into the leader role. These results show the relevance of providing leader mindfulness training.

Third, our results show that neuroticism plays a role in the association between mindfulness and relatedness need satisfaction: when neuroticism was high, or conversely when emotional stability was low, mindfulness had the largest impact on relatedness need satisfaction. This interaction between mindfulness and neuroticism shows that mindfulness may primarily exert its influence through an interaction with neuroticism, which is in line with the expectation that mindfulness primarily works through emotion regulation: when a highly neurotic leader is more able to open up for his/her emotions, rather than (over)react in the presence of subordinates or colleagues, it might be more easy to develop work relationships. The higher quality of these relationships will then help the leader to become more autonomously motivated and energized to be a transformational leader. Practically, these results indicate that leader neuroticism need not be a problem for leaders, if they learn how to deal with their nature and do not take their emotions out on the people around them. Mindfulness may be a valuable tool to achieve this. So again, providing mindfulness training, especially to leaders scoring high on neuroticism and low on trait mindfulness, might be a valuable intervention.

Finally, and more broadly speaking, mindfulness impacts employee well-being (Reb et al., 2014) as well as leader well-being and leadership (this study). The (emotion regulation) effect of mindfulness may therefore be important for all members of an organization. Consequently, we argue that embedding mindfulness in the culture of an organization may have the most beneficial effect. The research on collective mindfulness is only just emerging but shows promising results (Sutcliffe et al., 2016). When organizations invest in accepting presence, attention and thus mindfulness as a regular practice of leaders and employees, the enhanced well-being may lead organizations to excel, especially in a changing environment (Dane, 2011).

### Limitations and Future Research

Our research has several limitations that provide opportunities for future research. First of all, and most obviously, since this is a field survey study which used self-report data from leaders in several organizations at one time point, we cannot make actual causal inferences based on the results. Although this kind of data can be subject to bias, research shows that self-report data are not inherently flawed (Chan, 2009) and claims on common source bias are exaggerated (George and Pandey, 2017). Furthermore, we used apriori procedures to address common method bias (Podsakoff et al., 2003) as much as was possible within the constraints of the study design. Despite our efforts, the analyses indicated that common method bias may be present, thus the results from this study should be interpreted with caution. However, research does show that common method bias does not seem to be a threat when significant interactions are found (Evans, 1985): when common method variance is present, interactions can only be deflated (Siemsen et al., 2010). This indicates that common method variance might have suppressed part of our results. Nevertheless, we suggest that future studies replicate our findings with data from multiple sources (e.g., employees or the leaders' leaders) and multiple time points (longitudinal design), or in a laboratory setting to clearly establish causality. Based on our results and the possibility of common method bias, there is not only a pressing need for replication, but also for the use of more elaborate study designs. In a similar vein, we suggest that future research may want to use a more elaborate neuroticism scale as a replication tool (e.g., the original BFI with 44 items; Rammstedt and John, 2007), when the study design allows this, since we recognize that the internal consistency estimates in this study are quite low, even though the reliability and validity of two-item neuroticism measures has been established previously (Gosling et al., 2003; Rammstedt and John, 2007). In sum, a cross-sectional design based on self-reports has very clear limitations. We see our study, therefore, as a first exploration that should be interpreted with caution, but nevertheless may provide useful insights and directions for future research.

Second, while our study provides indications with regards to the importance of mindfulness and neuroticism for transformational leadership, we adopted a broad operationalization which did not take into account facets of mindfulness nor neuroticism. Faceted mindfulness questionnaires can be considered to provide more in-depth information with regards to which mindfulness facets influence psychological need satisfaction most. Useful questionnaires in this regard may be the Five Facet Mindfulness Questionnaire (Baer et al., 2008) or the Comprehensive Inventory of Mindfulness Experiences (CHIME; Bergomi et al., 2013). Future research may also want to use a faceted Big Five questionnaire to measure neuroticism (such as the NEO-PI-R; Costa and Mccrae, 1995). In this regard, research also showed that sub-facets of personality traits explained on average about twice as much variance in employee performance in comparison with aggregated traits (Judge et al., 2013). Therefore, future research can e.g., use personality questionnaires from Costa and Mccrae (1992,1995) to explain moderation effects of neuroticism in more detail. In addition, our results seem to support the self-regulatory hypothesis with regards to the effect of mindfulness, but we did not measure self-regulation itself. Rather, we investigated the interaction between mindfulness and neuroticism. Therefore, we should still be careful about the inferences we make with regards to mindfulness' emotion regulatory capacities. Future research may want to delve deeper into our results and measure emotion-regulation directly, e.g., through measuring established emotion-regulation strategies like surface acting (see e.g., Hülsheger et al., 2013).

Third, future research may also want to delve deeper into the results found in this study more generally: exactly how does mindfulness contribute to better relationships at work? Are there also other mindfulness-related mechanisms that play a part in this besides emotion regulation? Do mindful leaders communicate differently with their employees, which is then reflected in an enhanced relatedness need satisfaction? Perhaps leader relatedness need satisfaction based on mindfulness also has effects on the employee? Does the effect of mindfulness on leadership trickle down the organization from top leadership to the employee level? These and other interesting questions may be resolved through future research. It may for instance be very interesting to study whether mindfulness trickles down the organization and is able to have effects on other levels of the organization.

### Conclusion

Drawing mostly upon self-determination theory, integrated with a regulatory approach, our research uncovered the black box of leader-central antecedents of transformational leadership. The results indicate that psychological need satisfaction is the underlying mediating process in the relationship between leader mindfulness and transformational leadership. Moreover, neuroticism interacts with this effect for relatedness need satisfaction: when neuroticism is high, mindfulness has the highest impact on relatedness need satisfaction. Or conversely, when emotional stability is low, mindfulness has the lowest impact on relatedness need satisfaction. This is in line with research indicating that mindfulness might exert part of its influence through emotional regulation. Our research contributes both to theoretical developments integrating mindfulness in the leadership paradigm, while also offering suggestions for practice.

### Final Note

Our research reveals that leader mindfulness and leader psychological need satisfaction are relevant for transformational leadership. When organizations can support leader well-being and introduce mindfulness, positive effects can be expected for both the leader, as well as for his/her subordinates, perhaps especially when the leader scores high on neuroticism. It is our hope that future research will dig deeper into these findings, so that organizations can be persuaded to refocus on mindfulness and on the resulting well-being and psychological need satisfaction as a work relationship enhancer performance and performance booster.

## Ethics Statement

Our study was conducted in accordance with the ethical code of our university (University of Ghent). We obtained agreement from the directors of the elderly care homes prior to the data collection. All participants agreed voluntarily to participate in this study. The data were treated anonymously. Informed consents were signed by every participant. In addition, as we used paper-and-pencil questionnaires, the instructors were able to answer additional questions on e.g., the anonymization of data. Through this procedure, it was made sure that participants knew their rights and were able to withdraw at any moment in the study. This study did not induce psychological distress, nor involve a vulnerable population.

## Author Contributions

AnD was responsible the data collection design and overseeing the data collection, for the data analysis and for writing the manuscript. MA and AdD were responsible for the data collection design and overseeing the data collection, for reviewing the manuscript and for the final approval.

### Conflict of Interest Statement

The authors declare that the research was conducted in the absence of any commercial or financial relationships that could be construed as a potential conflict of interest.
