# Supplementary material for: When Mindfulness Interacts With Neuroticism to Enhance Transformational Leadership: The Role of Psychological Need Satisfaction
Source: Front Psychol. 2018 Dec 18;9:2588. doi: 10.3389/fpsyg.2018.02588 (PMC6305618; doi:10.3389/fpsyg.2018.02588)
Supplement: Supplementary file 1 [file Table_1.docx]

**Appendix 1**

|  |  | Mindfulness | Need satisfaction |  |  | Transformational leadership |
| --- | --- | --- | --- | --- | --- | --- |
|  |  |  | *Autonomy* | *Competence* | *Relatedness* |  |
| MF1* | I could be experiencing some emotion and not be conscious of it until some time later. *(R)** | .361 |  |  |  |  |
| MF2 | I break or spill things because of carelessness, not paying attention, or thinking of something else. *(R)* | .482 |  |  |  |  |
| MF3 | I find it difficult to stay focused on what’s happening in the present. *(R)* | .619 |  |  |  |  |
| MF4 | I tend to walk quickly to get where I’m going without paying attention to what I experience along the way. *(R)* | .682 |  |  |  |  |
| MF5 | I tend not to notice feelings of physical tension or discomfort until they really grab my attention. *(R)* | .420 |  |  |  |  |
| MF6 | I forget a person’s name almost as soon as I’ve been told it for the first time. *(R)* | .411 |  |  |  |  |
| MF7 | It seems I am “running on automatic”, without much awareness of what I’m doing. *(R)* | .685 |  |  |  |  |
| MF8 | I rush through activities without being really attentive to them. *(R)* | .662 |  |  |  |  |
| MF9 | I get so focused on the goal I want to achieve that I lose touch with what I’m doing right now to get there. *(R)* | .563 |  |  |  |  |
| MF10 | I do jobs or tasks automatically, without being aware of what I’m doing. *(R)* | .699 |  |  |  |  |
| MF11 | I find myself listening to someone with one ear, doing something else at the same time. *(R)* | .591 |  |  |  |  |
| MF12 | I drive places on “automatic pilot” and then wonder why I went there. *(R)* | .657 |  |  |  |  |
| MF13* | I find myself preoccupied with the future or the past.* *(R)* | .347 |  |  |  |  |
| MF14 | I find myself doing things without paying attention. *(R)* | .708 |  |  |  |  |
| MF15 | I snack without being aware that I’m eating. *(R)* | .445 |  |  |  |  |
| NS1 | I feel like I can be myself at my job |  | .702 |  |  |  |
| NS2 | At work, I often feel like I have to follow other people’s commands *(R)* |  | .579 |  |  |  |
| NS3 | If I could choose, I would do things at work differently *(R)* |  | .737 |  |  |  |
| NS4 | The tasks I have to do at work are in line with what I really want to do |  | .611 |  |  |  |
| NS5 | I feel free to do my job the way I think it could best be done |  | .658 |  |  |  |
| NS6 | In my job, I feel forced to do things I do not want to do *(R)* |  | .654 |  |  |  |
| NS7 | I don’t really feel competent in my job *(R)* |  |  | .565 |  |  |
| NS8 | I really master my tasks at my job |  |  | .778 |  |  |
| NS9 | I feel competent at my job |  |  | .904 |  |  |
| NS10 | I doubt whether I am able to execute my job properly *(R)* |  |  | .571 |  |  |
| NS11 | I am good at the things I do in my job |  |  | .857 |  |  |
| NS12 | I have the feeling that I can even accomplish the most difficult tasks at work |  |  | .767 |  |  |
| NS13 | I don’t really feel connected with other people at my job *(R)* |  |  |  | .701 |  |
| NS14 | At work, I feel part of a group |  |  |  | .601 |  |
| NS15 | I don’t really mix with other people at my job *(R)* |  |  |  | .667 |  |
| NS16 | At work, I can talk with people about things that really matter to me |  |  |  | .635 |  |
| NS17 | I often feel alone when I am with my colleagues *(R)* |  |  |  | .741 |  |
| NS18* | Some people I work with are close friends of mine* |  |  |  | .333 |  |
| MLQ1 | I make sure my nursing staff feels good when I am around. |  |  |  |  | .615 |
| MLQ2 | I use a few simple words to express what we can do. |  |  |  |  | .605 |
| MLQ3 | I help my nursing staff to think in new ways about old problems. |  |  |  |  | .766 |
| MLQ4 | I help my nursing staff to develop themselves. |  |  |  |  | .757 |
| MLQ5 | My nursing staff has complete faith in me. |  |  |  |  | .610 |
| MLQ6 | I draw my nursing staff a pleasant picture concerning all we can do. |  |  |  |  | .652 |
| MLQ7 | I provide my nursing staff with a fresh outlook on the matters. |  |  |  |  | .784 |
| MLQ8 | I let my nursing staff hear my opinion on how they are doing. |  |  |  |  | .635 |
| MLQ9 | My nursing staff is proud to be associated with me. |  |  |  |  | .556 |
| MLQ10 | I help my nursing staff to rethink existing ideas, which haven't been questioned before. |  |  |  |  | .668 |
| MLQ11 | I succeed to let my nursing staff rethink existing ideas, which haven't been questioned before. |  |  |  |  | .635 |
| MLQ12 | I attach personal importance to nursing staff feeling discouraged. |  |  |  |  | .581 |

*Items and factor loadings (principal axis factoring on one dimension); * = deleted item; (R) = reverse item*
